# Progression of Oropharyngeal Dysphagia in Amyotrophic Lateral Sclerosis: A Retrospective Cohort Study

**DOI:** 10.1007/s00455-021-10346-9

**Published:** 2021-07-23

**Authors:** Laura Mariani, Giovanni Ruoppolo, Armando Cilfone, Chiara Cocchi, Jacopo Preziosi Standoli, Lucia Longo, Marco Ceccanti, Antonio Greco, Maurizio Inghilleri

**Affiliations:** 1 Department of Sensorial Organs, Otorhinolaryngology Section, Sapienza University of Rome, Policlinico Umberto I, Viale dell’Università, 33, 00161 Rome, Italy; 2 Rare Neuromuscular Diseases Centre, Department of Human Neurosciences, Sapienza University of Rome, Policlinico Umberto I, Rome, Italy

**Keywords:** Dysphagia, Amyotrophic lateral sclerosis, FEES, Progression rate, Nutrition, Deglutition disorders, Deglutition

## Abstract

Little is known regarding the optimal timing of dysphagia assessment and PEG indication in amyotrophic lateral sclerosis (ALS). The study aims to investigate the progression of dysphagia in a cohort of ALS patients and to analyse whether there are variables linked to a faster progression of dysphagia and faster indication of PEG placement. A retrospective cohort study in 108 individuals with ALS. Fiberoptic endoscopic evaluation of swallowing was performed 6 monthly until PEG indication or death. Dysphagia severity and PEG indication were assessed using Penetration Aspiration Scale. Progression Index (PI) analysed the risk of disease progression (fast/slow) in relation to dysphagia onset and PEG indication. Patients were grouped based on ALS onset and PI. Person-time incidence rates were computed considering dysphagia onset and PEG indication from ALS symptoms during the entire observation period and have been reported as monthly and 6-month rates. Cox regression survival analysis assessed dysphagia and PEG risk factors depending on onset. Person-time incidence rates of dysphagia progression and PEG risk were increased based on type of ALS onset and PI. Patients with a fast progressing disease and with bulbar onset (BO) show statistically significant increased risk of dysphagia (BO 178.10% hazard ratio (HR) = 2.781 *P* < 0.01; fast 181.10% HR 2.811 *P* < 0.01). Regarding PEG risk, fast patients and patients with BO had a statistically significant increased risk (fast 147.40% HR 2.474 *P* < 0.01, BO 165.40% HR 2.654 *P* < 0.01). Fast PI predicts the likelihood of faster progression of dysphagia and PEG indication and should be included in multidisciplinary assessments and considered in the design of future guidelines regarding dysphagia management in ALS patients.

*Level of Evidence* Level IV.

## Introduction

Amyotrophic Lateral Sclerosis (ALS) is a neurodegenerative disorder characterized by upper and lower motor neuron degeneration, severe individual impairment with high impact in terms of disability-adjusted life years (DALYs) [1, 2]. It is estimated that the prevalence of ALS worldwide is 4.42 per 100,000 population and its incidence 1.59 per 100,000 [3]. Xu et al. reported significantly higher values in Western Europe (prevalence: 9.62, 95% CI 4.80–16.10; incidence: 2.76, 95% CI 2.00–3.64) and show a predilection in males (prevalence: 5.96, 95% CI 5.14–6.85) rather than in women (prevalence: 3.90, 95% CI 3.30–4.56) [3]. According to the European Federation of Neurological Societies (EFNS) guidelines on the Clinical Management of ALS (MALS) [1], the mean time from the onset of symptoms to diagnosis is 10–18 months. ALS patients have a very low survival rate: death generally occurs 2–4 years after onset and less than 10% survive beyond 10 years [4]. Patients typically die from respiratory complications, such as bronchopneumonia and aspiration/pneumonia events, which result from dysphagia due to bulbar sensory-motor neurodegeneration [1, 5, 6]. Bulbar impairment is a pathognomonic feature of ALS: patients develop speech and swallowing disorders that usually worsen with the progression of the disease, even though almost 30% display these symptoms from the onset of pathology [7]. Nowadays the most recommended approach considering ALS complexity is multidisciplinary care and its effects were recently investigated in comparison to general neurological care. Multidisciplinary care was found to be more effective at improving the survival rate of patients and this result was even greater for people with bulbar onset ALS [8]. Frequently, ALS patients develop an oropharyngeal impairment that concerns the tongue, but the underlying mechanisms of its motor dysfunction are not completely understood. Nevertheless, the tongue impairment seems to represent a major risk factor for aspiration [9]. The occurrence of dysphagia in ALS patients is also related to impairments in the upper aero digestive tract, respiratory and laryngeal muscles to the extent that they affect the expiratory phase of voluntary cough. Recently, Plowman et al. underlined the strong connection between poor effective voluntary cough and the presence of penetration/aspiration events [10]. Trained experts on swallowing such as ENT laryngologists or speech and language pathologists (SLP) should therefore search for and recognize the presence of dysphagia as early as possible and prevent its complications whenever possible [11, 12]. A comprehensive swallowing evaluation based on clinical and instrumental confirmation as well as self-reported symptoms should be conducted. The Northeast ALS (NEALS) bulbar subcommittee recently recommended that direct visualization of swallowing function and consequent evaluation of swallowing performance in terms of safety and efficacy should be assessed in ALS patients through the use of instrumental techniques by appropriately trained ENT laryngologists or SLP [11]. Videofluoroscopic Swallowing Study (VFSS) is often considered the reference standard for evaluating dysphagia, although recent studies suggest that Fiberoptic Endoscopic Evaluation of Swallowing (FEES) is a valid, repeatable and low-cost alternative, able to evaluate pharyngo-laryngeal sensitivity in addition to motility [12, 13]. In accordance with EFSN guidelines [1], the initial management of dysphagia should be based on modification of food texture and density, taking great care over the education and training of ALS patients and caregivers in feeding and swallowing techniques. With the worsening of dysphagia and considering weight loss, respiratory function and overall condition, tube feeding is often recommended. Percutaneous endoscopic gastrostomy (PEG) is the most commonly used procedure for enteral nutrition in ALS, but percutaneous radiologic gastrostomy (PRG) is also considered a suitable alternative (1). In spite of increasing clinical interest in dysphagia in ALS patients, discrepancies related to its assessment and management are still evident [11, 14]. The timing of referral for laryngological evaluation varies and should depend on the presence and progression of clinical signs [15]. A dysphagia assessment should be performed in all patients with ALS, both at diagnosis and during follow up, with a recommended frequency of every 3 months, as part of a complete clinical and neurological evaluation [15]. Additionally, the timing of PEG/PRG recommendations also varies. The conclusion is that a multi-layered decision-making process that is tailored to the patient must be adopted. However, clinicians should be aware that postponing PEG to a late disease stage might increase the risk of the procedure failing mainly due to infections, secondary to poor nutritional status, advanced age, comorbidities, and impairment of the immune apparatus [1]. Consequently, our study was designed to retrospectively analyse the progression of oropharyngeal dysphagia in ALS, considering both patients with bulbar and spinal onset. Our primary aim was to investigate the progression of dysphagia in a cohort of ALS patients. Our secondary aim was to better understand variables related to faster progression of dysphagia and PEG positioning.

## Materials and Methods

### Study Design and Sample Groups

A retrospective cohort study on patients with ALS evaluated for dysphagia with an assessment period from 2005 to 2018 at the Rare Neuromuscular Diseases Centre of the Department of Human Neurosciences of the Policlinico Universitario Umberto I, Rome. From the whole database of 380 patients we collected complete data in 108 patients (58 females and 50 males; mean age at ALS diagnosis 66.95 ± 10.42) who were diagnosed with swallowing impairment during the follow up at FEES evaluation. Inclusion criteria was clinically defined ALS according to the revised El Escorial criteria (r-EEC) [16]. Exclusion criteria were as follows: controversial ALS diagnosis, incomplete medical history, no clinically or endoscopic-based diagnosis of dysphagia due to loss of data, for example, sudden death prior to development of dysphagia, change of neurological centre for treatment or becoming housebound due to their deteriorating condition. Data on individual patients were collected and analysed, starting with their first neurological assessment (T0). All of the data concerning the ENT laryngological evaluations, which were carried out every 6 months, was included until PEG indication or death, in order to establish the dysphagia progression rate. The medical history of all patients and in particular when ALS symptoms started, ALS onset type, time of ALS diagnosis, timing of dysphagia onset and PEG indication were collated. Additionally, the sample was divided into 2 groups depending on the type of onset: bulbar onset (BO) or spinal onset (SO).

### Dysphagia Evaluation

The standard methodology adopted by our multidisciplinary medical team provides each patient who has clinically definite or suspected ALS an evaluation by an ENT laryngologist through FEES. This happens at first access to the neurological centre and then every 3 or 6 months thereafter depending on the patient's needs and separately from complaining of swallowing disorders. Each patient underwent FEES, performed by two expert ENT laryngologists using a flexible fiberscope (EF-N XION Nasopharingoskope, Germany). Each patient was placed in a seated or semi-seated position. To begin with two bolus of 5 ml soft liquid (yoghurt) were administered and then bolus of < 5 ml, 5 ml and 10 ml of thin liquid (milk) whilst asking the patient to hold the bolus in their oral cavity and then each time to swallow upon the command of the operator according to the Langmore procedure [17]. Each patient was assessed by both ENT laryngologists through a video endoscopy camera (MediCam Plus LT, Inventis) connected to a laptop (3rd generation Intel® Quad Core i7).

### Dysphagia Scoring

Based on FEES, each patient was given a score according to the Penetration Aspiration Scale (PAS) [18] and each outcome was recorded in the medical records of the patient. The evaluation is scored on an 8-point scale and is routinely performed in the clinic and used to characterize depth and response to airway invasion during swallowing. In order to analyse the presence/absence of penetration/aspiration at first FEES evaluation (T0), the sample was divided into three groups according to the PAS scale (absence of penetration/aspiration, score 1; presence of penetration, score 2–5; and presence of aspiration, score 6–8). The cut off point for the diagnosis of dysphagia was considered PAS ≥ 3. The cut off point for PEG indication was considered PAS ≥ 6. Indeed, the indication for PEG placement was undertaken when the risk of aspiration was evident and urgent measures for alternative feeding had to be initiated to prevent the patient from developing further complications.

### Progression Index

The progression index (PI) [19], an index predictor of survival, was used to analyse the risk of disease progression in relation to the onset of dysphagia and for PEG indication. PI is a Δ score that was computed considering the reduction in Amyotrophic Lateral Sclerosis Functioning Rating Scale Revisited (ALSFRS-R) [20, 21] score from the beginning of symptoms to baseline. PI = (Total ALSFRS-R score possible (48)—first assessment ALSFRS-R score of patient; divided by the number of months from the patient's initial symptoms onset). ALSFRS-R scale is routinely performed by the clinic neurologist for every assessment. The baseline score at first neurological assessment was documented from the medical records of the patient and patients were then separated on the basis of their rate of disease progression: patients with a slow progressing disease (pSlow) had PI ≤ 0.5; patients with a fast progressing disease (pFast) had PI > 0.5 [19]. Finally, the pSlow and pFast patients were then further divided depending on onset in BO/pSlow, BO/pFast, SO/pSlow and SO/pFast.

### Statistical Analysis

Shapiro–Wilk test was used to assess normal data distribution. Categorical variables were calculated using frequencies and proportions whilst continuous data were estimated by means, standard deviations and ranges. Person-time incidence rates were computed considering dysphagia onset and PEG indication from ALS symptoms during the entire observation period and have been reported as monthly and six-month rates. Cox regression survival analysis was performed for the whole sample (*N* = 108) to assess the differences in dysphagia risk factors whilst considering, as a dependent variable, the time from the onset of ALS symptoms to primary onset of dysphagia and to PEG indication. Adjustments were made for the effects of the five covariates found to be predictive in the survival model: age at ALS symptom onset, age at ALS diagnosis, bulbar/spinal onset, slow/fast progressors and gender. Calculated p values were 2-sided, a *P*-value of less than 0.05 was considered as significant and the range of confidence interval (CI) was 95%, where appropriate. Statistical Analysis was performed using The Statistical Package for Social Sciences (SPSS) ver. 25 (SPSS IBM).

## Results

### Descriptive Analysis

The sample consisted of 68 patients with bulbar onset (BO) and 40 patients with spinal onset (SO), and using the calculated PI, 63 patients were pFast (40 BO and 23 SO) and 45 pSlow (28 BO and 17 SO). The estimated mean time for the complete sample between symptom onset and ALS diagnosis time was 13.8 ± 9.5; range 2–48 months. The mean follow up time was 15.8 ± 12.2; range 0–60 months. The mean time for dysphagia onset from ALS symptoms was 20.9 ± 15.1; range 2–96 months and the mean time to PEG indication from ALS symptoms was 25.7 ± 16.8; range 0–71 months. At the first FEES examination (T0), 27 patients (25%) had no penetration/aspiration, 77 (71.3%) had penetration, and only 4 patients (3.7%) had aspiration. Overall 44 patients (47.5%) already had PAS ≥ 3 at T0 and 64 (52.5%) developed dysphagia during the follow-up. Tables 1 and 2 show the descriptive analysis of all the study groups and subgroups. All patients in the sample experienced dysphagia onset, but only 87 achieved PEG indication, whilst 21 patients were not included in follow up due to death.

**Table 1:** ALS patients descriptive analysis

	Bulbar onset	Spinal onset	pFast	pSlow	Overall
	Total	Total	Total	Total	Total
N	68 (63%)^b^	40 (37%)	63 (58.3%)	45 (41.7%)	108
Age at T0 (first examination)	66.6 ± 9.4 (41 to 80)	64.1 ± 12 (40 to 82)	67.1 ± 9.6 (40 to 82)	63.8 ± 11.3 (41 to 82)	65.75 ± 10.49 (40 to 82)
Age at ALS symptom onset	68.7 ± 9.8 (45 to 84)^c^	63.9 ± 10.9 (41 to 83)	68.2 ± 10.4 (41 to 84)	65.2 ± 10.3 (42 to 81)	66.95 ± 10.43 (41 to 84)
Mean Follow up^a^	12.7 ± 9.4 (0 to 42)	21.1 ± 14.5 (6 to 60)	13.5 ± 9.3 (0 to 42)	19.1 ± 14.9 (0 to 60)	15.83 ± 12.2 (0 to 60)
Time from symptom onset to dysphagia event^a^	15.68 ± 10.19 (3 to 49)	29.83 ± 17.90 (2 to 96)	15.29 ± 9.67 (2 to 47)	28.8 ± 17.77 (6 to 96)	20.92 ± 15.13 (2 to 96)
Time from symptom onset to PEG event^a^	20.69 ± 13.48 (0 to 63)	34.43 ± 16.61 (6 to 61)	20.41 ± 12.64 (0 to 53)	33.29 ± 17.47 (0 to 71)	25.78 ± 16.08 (0 to 71)
No penetration/aspiration in FEES at T0	8 (11.8%)	19 (47.5%)	9 (14.3%)	18 (40%)	27 (25%)
Penetration in FEES at T0	56 (82.4%)	21 (52.5%)	51 (81%)	26 (57.8%)	77 (71.3%)
Aspiration in FEES at T0	4 (5.9%)	0	3 (4.8%)	1 (2.2%)	4 (3.7%)

^a^Months

^b^Categorical variables are reported as Number and (Percentage)

^c^Continuous data are shown by means, standard deviations and (ranges)

**Table 2:** ALS Onset/PI subgroups descriptive analysis

	BO/pFast	BO/pSlow	SO/pFast	SO/pSlow
	Total	Total	Total	Total
N	40 (58.8%)^b^	28 (41.2%)	23 (57.5%)	17 (42.5%)
Age at T0 (first examination)	68.2 ± 7.5 (52 to 82)^c^	63.9 ± 11.3 (41 to 80)	64.5 ± 12.4 (40 to 80)	63.6 ± 11.9 (51 to 82)
Age at ALS symptom onset	69.2 ± 9.9 (45 to 84)	68.1 ± 11.3 (46 to 81)	66.6 ± 11.2 (41 to 83)	60.3 ± 9.7 (42 to 79)
Mean Follow up^a^	11.85 ± 9.2 (0 to42)	13.9 ± 9.8 (0 to 36)	16.4 ± 9.1 (6 to 36)	27.5 ± 18.1 (6 to 60)
Time from symptom onset to dysphagia event^a^	9.75 ± 7.3 (2 to 36)	14.5 ± 6.7 (5 to 30)	12.5 ± 10.3 (2 to 47)	24.5 ± 9.2 (11 to 48)
Time from symptom onset to PEG event^a^	17.1 ± 11.4 (0 to 48)	25.9 ± 14.7 (0 to 63)	26.3 ± 12.8 (6 to 53)	45.5 ± 14.8 (18 to 71)
No penetration/aspiration in FEES at T0	3 (7.5%)	5 (17.9%)	6 (26.1%)	13 (76.5%)
Penetration in FEES at T0	34 (85%)	22 (78.6%)	17 (73.9%)	4 (23.5%)
Aspiration in FEES at T0	3 (7.5%)	1 (3.6%)	0	0
Six-month person-time incidence of dysphagia onset	61.54%	41.28%	48.08%	24.52%
Six-month person-time incidence of PEG indication	28.15%	20.69%	16.89%	10.09%

^a^Months

^b^Categorical variables are reported as Number and (Percentage)

^c^Continuous data are shown by means, standard deviations and (ranges)

### Dysphagia and PEG Person-Time Incidence Rates

With regard to the timing of dysphagia onset, patients with BO and pFast had 8.53% and 9.31% monthly person-time incidence rates from symptom onset, respectively. Conversely, patients with SO and pSlow had lower percentages 5.49% and 5.47%. The six-monthly person-time incidence rates were 51.19% for patients with BO, 34.14% for patients with SO, 55.83% for pFast and 32.81% for pSlow. Patients with BO and pFast also had higher monthly person-time incidence rates for PEG indication from symptom onset at 4.29% and 4.36%, respectively, compared to patients with SO (2.64%) and pSlow (2.83%). The PEG indication six-monthly person-time incidence rates were 25.73% for patients with BO, 15.83% for patients with SO, 26.16% for pFast and 16.99% for pSlow. Rates for BO/pFast, BO/pSlow, SO/pFast and SO/pSlow are reported in Table 2.

### Cox Regression Survival Analysis

**Fig. 1:**
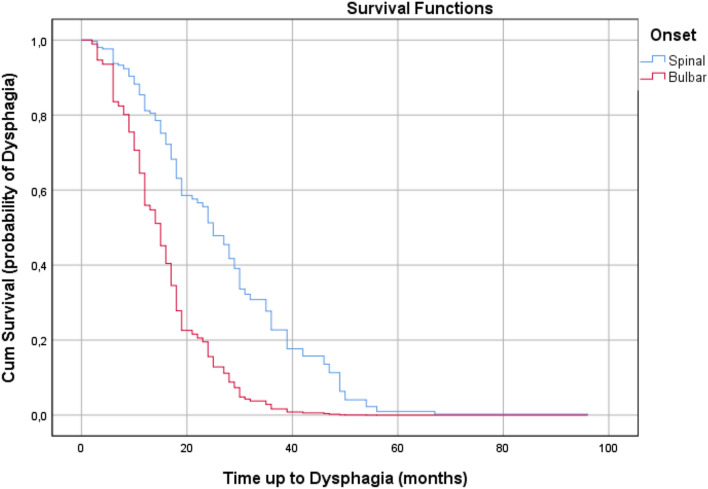
Survival plot related to occurrence of dysphagia in patients during observation period. Censored observations are not reported

**Fig. 2:**
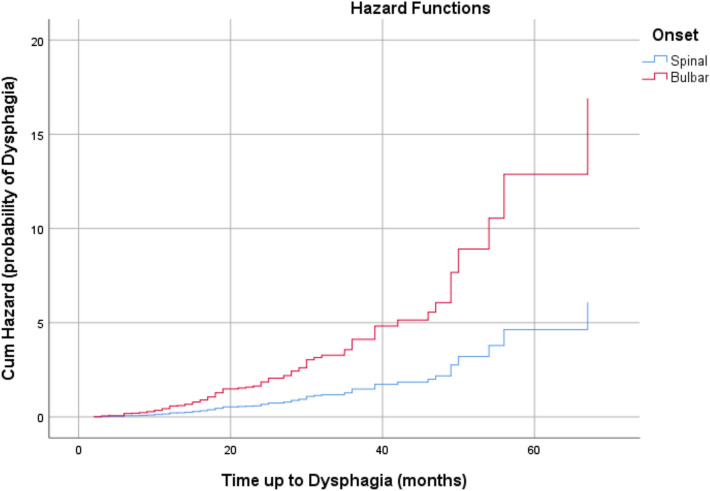
Cumulative Hazard plot related to occurrence of dysphagia in patients during observation period. Censored observations are not reported

In the Cox regression survival analysis regarding the onset of dysphagia (*N* = 108), likelihood ratio tests resulted in statistically significant *P*-values (*P* < 0.01 and *P* < 0.01, respectively) after adjustment for the five covariates. All of the covariates, except gender, age at ALS symptom onset and age at ALS diagnosis, predicted survival time at = 0.01: 1.023[bulbar/spinal (bulbar)] + 1.034[slow/fast (fast)]. Table 3 shows regression coefficients, standard error, *P* values, and hazard ratios for each covariate. pFast had an increased risk of dysphagia onset at 181.10% each month (hazard ratio (HR) 2.811) whilst patients with BO had an increased risk at 178.10% each month (hazard ratio (HR) 2.781). At the mean of covariates, the 20-month survival rates in patients with BO were about 20% and slightly less than 60% of that for patients with SO (Figs. 1, 2).

**Table 3:** Cox regression survival analysis based on dysphagia

	Regression coefficients*	SE^d^	*P*-value	Hazard Ratio	CI 95% HR	Percentage
Gender (female)^c^	0.217**	0.204	0.288	1.243	0.832–1.855	24.30
Age at T0	0.004	0.01	0.702	1.004	0.985–1.023	0.40
Slow/Fast (fast)^b^	1.034	0.221	0.000001	2.811	1.821–4.338	181.10
Age at ALS symptom onset	− 0.001	0.01	0.958	0.999	0.980–1.855	− 0.10
Onset bulbar/spinal (bulbar)^a^	1.023	0.227	0.00001	2.781	1.782–4.340	178.10

*A positive sign means that the hazard (risk of dysphagia or PEG) is higher, subjects with higher values of this variable had worse prognosis

**A positive sign indicates that be female increases the hazard risk of dysphagia (lower survival rates) by a factor of 1.243 or 24.3%

***Hazard ratio give the effect size of covariates. Having a bulbar onset (onset = 1) increases the hazard by a factor of 2.781 or 178.1%

****Standard error

^a^Bulbar/Spinal: spinal = 0; bulbar = 1

^b^Slow/Fast: slow = 0; fast = 1

^c^Male/Female: male = 0; female = 1

**Fig. 3:**
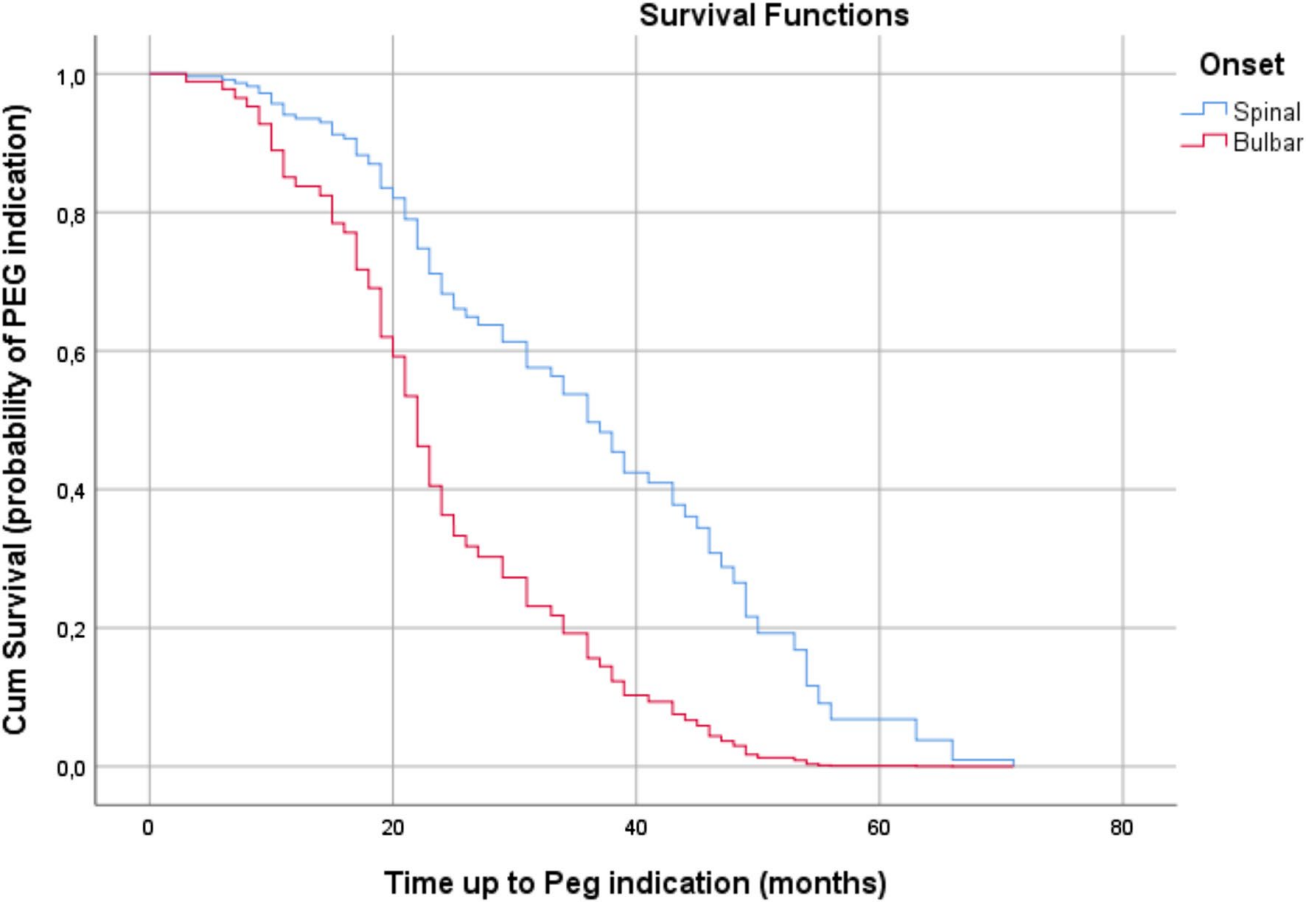
Survival plot related to occurrence of PEG indication in patients during observation period. Censored observations are not reported

**Fig. 4:**
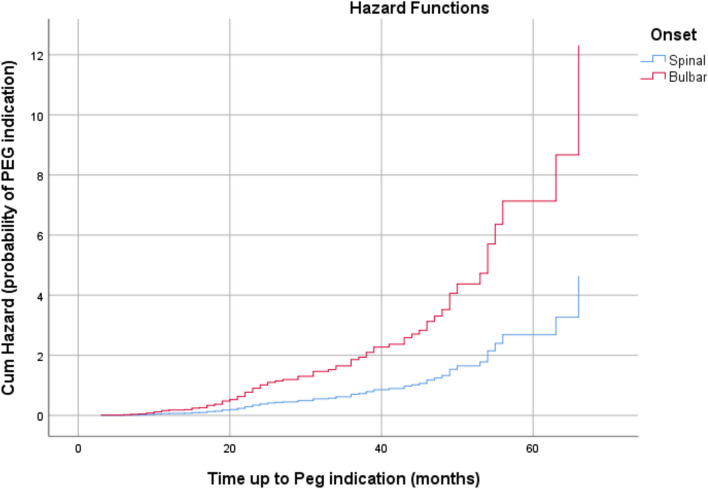
Cumulative Hazard plot related to occurrence of PEG indication in patients during observation period. Censored observations are not reported

Likelihood-ratio test resulted in statistically significant outcomes (*P* < 0.01) regarding analysis of expected survival rates regarding PEG indication. In this model Onset and slow/fast covariates predicted survival times at = 0.01 RISK = 0.976[bulbar/spinal (bulbar)] + 0.906[slow/fast (fast)]. Table 4 shows regression coefficients, standard error, *P*-values, and hazard ratios for each covariate. pFast, on a cumulative monthly basis, had an increased risk of PEG indication at 147.40% (HR 2.474) and patients with BO had an increased risk at 165.40% each month (hazard ratio (HR) 2.654). In patients with BO the 20-month survival rate was about 60% and about 80% for patients with SO (Fig. 3), whereas the cumulative risk that a patient with BO will develop indication to PEG was slightly under 1.00, whilst in patients with SO it was about 0.40 at the 20-month point of observation from the onset of ALS symptoms (Fig. 4).

**Table 4:** Cox regression survival analysis based on PEG indication

	Regression coefficients*	SE****	*P*-value	Hazard Ratio	CI 95% HR	Percentage
Gender (female)^c^	0.025**	0.23	0.913	1.025	0.654–1.608	2.50
Age at T0	0.006	0.011	0.560	1.006	0.986–1.027	0.60
Slow/fast (fast)^b^	0.906	0.246	0.000001	2.474	1.527–4.009	147.40
Age at ALS symptom onset	0.007	0.012	0.559	1.007	0.984–1.031	0.70
Onset Bulbar/Spinal (bulbar)^a^	0.976	0.246	0.000001	2.654	1.618–4.354	165.40

*A positive sign means that the hazard (risk of dysphagia or PEG) is higher, subjects with higher values of this variable had worse prognosis

**A positive sign indicates that be female increases the hazard risk of PEG indication (lower survival rates) by a factor of 1.025 or 2.5%

***Hazard ratio give the effect size of covariates. Having a bulbar onset (onset = 1) increases the hazard by a factor of 2.654 or 165.4%

****Standard error

^a^Bulbar/Spinal: spinal = 0; bulbar = 1

^b^Slow/Fast: slow = 0; fast = 1

^c^Male/Female: male = 0; female = 1

## Discussion

The progression of dysphagia in individuals with ALS has not been fully described in the literature. There are many reasons for this including minimal data studies, low life expectancy [22, 23] and the unpredictability and differences in clinical progression of ALS in bulbar and spinal onset [4, 24]. A series of factors are described in the literature affecting the prognosis of ALS; such as older age [24, 25], bulbar onset [4, 5, 24, 26], progression rate of ALSFRS-R score [4, 12, 13, 19, 24, 26], nutritional status [4, 27] and respiratory status with lower forced vital capacity (FVC%) at diagnosis [4, 28, 29]. We believe that the traditional stratification used for clinical trials of ALS patients in bulbar and spinal onset is no longer sufficient or adequate [4] and for this reason, as in our study, we decided to evaluate patients using PI [19]. To date, as far as we are aware, this is the first study that analyses dysphagia evolution in a large cohort of patients with ALS during the course of the disease. Consistent with MALS [1] the mean time from the onset of symptoms to diagnosis was 13.8 ± 9.5 months. These results, combined with the data collected at the onset of dysphagia, are relevant to and should be featured in clinical practice because often the diagnosis of ALS only occurs at the first neurological and laryngological examination. Most of the patients examined at T0 already had mild or moderate impairment of swallowing, especially patients with bulbar onset or pFast. For this reason, during the patient’s initial examination it is critical that swallowing disorders are promptly dealt with by trained experts on swallowing as an important part of the multidisciplinary evaluation of the patient in cases where the presence of difficulty in swallowing is suspected. In particular, involuntary weight loss, coughing or choking during meals, length of time needed to eat, saliva management, speaking rate, and slurring of speech need to be assessed [11]. Our data show that patients with BO have a shorter time from the beginning of symptoms to the onset of dysphagia and, in fact, at the first laryngological examination 88.3% already had impaired swallowing. On the other hand, patients with SO already had a swallowing disorder present in 52.5% of cases. Patients with BO and pFast had a higher percentage (92.5%) of patients with swallowing already compromised at the first laryngological evaluation. However, most of them (85%) did not experience aspiration at FEES therefore suggesting preliminary impairment only during the oral phase and also preservation of laryngeal sensitivity [5]. Alternatively, BO/pSlow and SO/pFast, achieved similar percentages in dysphagic patients at first evaluation demonstrating a higher impact of PI rather than onset alone. The person-time rate at which new cases of dysphagia occurred in the sample examined every month and every 6 months was greater for patients with BO than for patients with SO and was also greater for BO/pFast and SO/pFast compared to BO/pSlow and SO/pSlow. These data confirm that, in addition to early management, it is necessary to establish serial controls over time that should be weighted to the patient's clinical features. Following EFNS guidelines [1], patients should be reviewed every 2–3 months, although they may require more frequent reviews in the months following diagnosis or in the latter stages of disease, and less frequently reviewed if their disease is progressing slowly. Our data clearly show that patients with BO/pSlow and with SO/pFast had a comparable 3-month incidence of personal dysphagia of 20.60% and 24.04%, respectively. Therefore they need to be followed up more frequently than 3 month intervals, in particular during the months immediately following diagnosis, compared to patients with SO/pSlow, whose follow-up intervals could remain at every 3 months. In clinical practice PEG is generally recommended according to symptoms, nutritional status and respiratory function and should be performed before vital capacity falls below 50% of predicted levels [1, 30, 31]. Early recognition of dysphagia, possibly when performing a periodic objective swallowing evaluation in ALS patients, allows for the identification of patients who need PEG, given that 70% of patients who have used PEG have a higher probability of survival [6]. Our data show that average time from symptom onset to PEG indication was shorter in BO/pFast and that patients with these features had a PEG recommendation around 24 months from symptom onset, one year earlier than BO/pSlow and SO/pFast patients who had similar percentages. In our survival analysis, we found that neither age at ALS onset nor at ALS diagnosis or gender were associated with increased risk of dysphagia or PEG. By contrast, a study [25] of 33 patients found a proportional relationship between age at ALS symptoms onset and both the timing of worsened swallowing functionality, the need for non-oral feeding and higher risk in women. Further research should be conducted to understand whether gender and age can be considered a risk factor for dysphagia onset and PEG. Indeed, our study demonstrated that fast PI is a statistically significant risk factor in both patients with SO and BO, which needs to be considered at the beginning of the patient's evaluation. Multidisciplinary management is crucial for ALS patients since they have a life expectancy of only 3–5 years and whilst dysphagia always occurs the timing and severity differs for each patient. According to a further study [32] which recommends a VFSS every 6 months after bulbar symptom onset with a one year follow up to evaluate tube feeding, our data suggest that 12 months is a clear cut-off point for dysphagia onset in BO/pFast, BO/pSlow and SO/pFast patients. Nevertheless, in our opinion, swallowing evaluations should be more frequent. More importantly, a delay in laryngologist or SLP referrals after the beginning of symptoms might lead to the risk of malnutrition or pulmonary complications in ALS patients. In fact it has been shown that active and aggressive multidisciplinary management enhances prognosis, particularly amongst patients who have ALS with bulbar dysfunction [33].

## Conclusions

In conclusion, using retrospective data collected over a 13-year period, we have shown that the timing of laryngological referral depends on clinical features in ALS patients. In particular, according to PI, pFast patients need several evaluations even if they have spinal onset. PEG indication needs to be considered empirically after 1 year from ALS symptoms onset in BO/pFast, around 2 years in BO/pSlow and SO/pFast patients and after 3 years in SO/pSlow. Age does not seem to be associated with increased risk of dysphagia onset or the need for non-oral feeding. It is clear that gender risk needs further research. Most importantly, our research suggests that PI should be included in multidisciplinary patient assessments and we therefore recommend that careful consideration of these findings be incorporated into the design of future guidelines regarding dysphagia management in ALS patients.
